# Downregulation of miR-497-5p Improves Sepsis-Induced Acute Lung Injury by Targeting IL2RB

**DOI:** 10.1155/2021/6624702

**Published:** 2021-04-12

**Authors:** Wei Lou, Jieping Yan, Weisi Wang

**Affiliations:** ^1^Department of Respiratory Medicine, Zhuji People's Hospital of Zhejiang Province, Zhuji Affiliated Hospital of Shaoxing University, 9 Jianmin Road, Taozhu Street, Zhuji, Zhejiang, 311800, China; ^2^Department of Pharmacy, Zhejiang Provincial People's Hospital, People's Hospital of Hangzhou Medical College, 158 Shangtang Road, Hangzhou 310014, China; ^3^Department of Respiratory and Critical Care Medicine, The Second Affiliated Hospital of Zhejiang Chinese Medical University, 318 Chaowang Road, Hangzhou, Zhejiang 310005, China

## Abstract

**Introduction:**

Acute lung injury (ALI) induced by sepsis is a process related to inflammatory reactions, which involves lung cell apoptosis and production of inflammatory cytokine. Here, lipopolysaccharide (LPS) was applied to stimulate the mouse or human normal lung epithelial cell line (BEAS-2B) to construct a sepsis model *in vivo* and *in vitro*, and we also investigated the effect of miR-497-5p on sepsis-induced ALI. *Material and Methods*. Before LPS treatment, miR-497-5p antagomir was injected intravenously into mice to inhibit miR-497-5p expression *in vivo*. Similarly, miR-497-5p was knocked down in BEAS-2B cells. Luciferase reporter assay was applied to predict and confirm the miR-497-5p target gene. Cell viability, apoptosis, the levels of miR-497-5p, IL2RB, SP1, inflammatory cytokine, and lung injury were assessed.

**Results:**

In BEAS-2B cells, a significant increase of apoptosis and inflammatory cytokine was shown after LPS stimulation. In septic mice, increased inflammatory cytokine production and apoptosis in lung cells and pulmonary morphological abnormalities were shown. The miR-497-5p inhibitor transfection showed antiapoptotic and anti-inflammatory effects on BEAS-2B cells upon LPS stimulation. In septic mice, the miR-497-5p antagomir injection also alleviated ALI, apoptosis, and inflammation caused by sepsis. The downregulation of IL2RB in BEAS-2B cells reversed the protective effects of the miR-497-5p inhibitor against ALI.

**Conclusion:**

In conclusion, downregulation of miR-497-5p reduced ALI caused by sepsis through targeting IL2RB, indicating the potential effect of miR-497-5p for improving ALI caused by sepsis.

## 1. Introduction

Sepsis is one of the most complex systemic inflammatory response syndromes caused by infection, which cause multiple organ dysfunction and eventually lead to death [1, 2]. The lung is actually the first organ to respond to sepsis [3]. Acute lung injury (ALI) induced by severe sepsis [4] is actually lung cell apoptosis caused by inflammation [5]. And studies found that ALI caused by sepsis led to a higher mortality rate [6]. However, there is currently no available pharmacologic therapy that can reduce the mortality rate of ALI patients. Therefore, it is essential to find the effective treatment of ALI.

miRNAs refer to small noncoding RNAs that are involved in the regulation of cell development, immunity, proliferation, metabolism, and apoptosis [7]. Recent works have demonstrated that miRNAs are devoted to both inflammation-induced apoptosis and sepsis-induced ALI processes [8]. The knockdown of miR-199a served to protect lung tissue from acute respiratory distress syndrome caused by sepsis through inhibiting excessive inflammation [9]. miR-145 reduced sepsis-induced lung injury by suppressingTGFBR2 signal transduction [10]. Therefore, the study for the regulatory role of miRNA in sepsis-induced ALI is of vital importance.

miR-497-5p was a member of the miR-15/16 family [11]. And miR-15a and miR-16 of the miR-15/16 family were verified to be upregulated in the serum of neonatal sepsis patients and inhibited inflammation induced by lipopolysaccharide (LPS) [12]. Growing evidence had shown that miR-497-5p was involved in melanoma [13], gastric cancer [14], and esophageal squamous cell carcinoma and hepatocellular carcinoma [15, 16]. Besides, it was reported that miR-497-5p regulated inflammation-related signaling pathways in hepatocellular carcinoma [17]. However, the correlation between miR-497-5p in ALI induced by sepsis is unclear.

IL-2 is essential for regulating immune response, and IL2RB is a subunit of the IL-2 receptor (IL-2R). As an inflammation-related gene [18], the absence of IL2RB in humans or mice leads to immune disorder [19]. Studies had reported that IL2RB played a role in inflammatory diseases such as lung cancer [20, 21], atopic dermatitis [22], and steatohepatitis [23]. Also, IL2RB was negatively correlated with Sequential Organ Failure Assessment (SOFA) and mortality of sepsis [24]. However, the role of IL2RB in the regulatory mechanism of ALI caused by sepsis is unclear.

In this research, we demonstrated that low expression of miR-497-5p exerted a crucial role in protecting ALI caused by sepsis. Additionally, low expression of miR-497-5p had been shown to protect lung tissue by targeting upregulation of IL2RB. These findings supported the idea that the potential treatment capacity of miR-497-5p in ALI is caused by sepsis and provided a potential novel strategy for treating ALI induced by sepsis.

## 2. Materials and Methods

### 2.1. Data Acquisition and Bioinformatics Analysis

The miRNA (normal: 8; sepsis: 10) and mRNA (normal: 20; sepsis: 10) expression profiles related to sepsis were from the GEO database, and the Limma package was applied to analyze the differential (|logFC | >2, padj < 0.05 were set as the threshold). The target miRNA was then targeted and predicted by FunRich (version 3.13), and the target gene was obtained by taking the intersection with the differential mRNAs. The transcription factor that regulated the target miRNA was predicted, and the target miRNA promoter region (2000 bp before the transcription start position) and the binding sequences with the transcription factor were predicted.

### 2.2. Sepsis-Induced Acute Lung Injury Mouse Model

Experiments were conducted under the guidelines of the Administration of Animal Experiments for Medical Research Purposes and Animal Ethics. Adult male C57BL/6 mice (28-32 g) were applied as experimental models. After the mice were fixed on the operating table, 1% pentobarbital 5-6 ml/kg was intraperitoneally injected into mice for anesthesia. The neck was cut longitudinally in the middle to expose the trachea. LPS (4 ml/kg, Sigma-Aldrich) was injected into the trachea and absorbed fully when the mice were shaken, and then the incision was sutured. Lung tissues and blood of sacrificed mice were collected for subsequent detection. Mice were divided into 4 groups randomly: sham-operation group, LPS group (sepsis model group), LPS+miR-497-5p antagomir group, and LPS+NC antagomir group. The tail vein injection of miR-497-5p antagomir or NC antagomir (GenePharma) was performed 15 min before LPS treatment.

### 2.3. H&E Staining

After being fixated in 4% paraformaldehyde, dehydrated, and paraffin embedded, the tissue was sliced into 5 *μ*m thick sections. After hydrating by xylene, the sections were immersed in anhydrous ethanol. Then, hematoxylin and eosin (H&E) was used to stain the sections [25]. The degree of lung injury was assessed by researchers blinded to the experiment.

### 2.4. Cell Culture and Transfection

The human normal lung epithelial cell line BEAS-2B (cell bank of Chinese Academy of Sciences of China) was cultured in DMEM (Gibco) containing 10% heat-inactivated FBS (Gibco) in a 37°C humidified atmosphere with 5% CO_2_. miR-497-5p mimic, miR-497-5p inhibitor, si-SP1, pcDNA3.1-SP1, si-IL2RB, and corresponding control were synthesized and transfected into BEAS-2B cells by Lipofectamine 2000 (Invitrogen). Cells were stimulated with LPS for constructing an *in vitro* model after transfection.

### 2.5. Luciferase Reporter Analysis

For miRNA target gene validation, the IL2RB 3′-UTR fragment with the miR-497-5p putative binding site was cloned the luciferase gene downstream in pmirGlo vector (GenePharma). The mutant IL2RB 3′-UTR was applied to build a mut vector of IL2RB. Cells were treated with miR-497-5p mimic (or NC mimic) and IL2RB-wt (or IL2RB-mut). For miRNA transcription factor validation, SP1 putative binding sites on the miR-497-5p promoter region (BS1 and BS2) cloned the luciferase gene downstream in the pmirGlo vector. Cells were treated with pcDNA3.1-SP1 (or pcDNA3.1) and BS1-wt (or BS1-mut) or BS2-wt (or BS2-mut). Then, luciferase activity was examined.

### 2.6. Quantitative Real-Time PCR (qRT-PCR)

Firstly, TRIzol (Beyotime, Shanghai) was devoted to extract total RNA from tissues and cells based on the manufacturer's recommendation. Then, a one-step miRNA RT kit (Haigene, Harbin) or BeyoRT™ First Strand cDNA Synthesis Kit (Beyotime, Shanghai) was used to reversely transcribe complementary DNA (cDNA) for miRNAs or mRNAs, respectively. Next, a Hieff Unicon TaqMan multiplex qPCR master mix (Yeasen, Shanghai) was applied to examine the levels of RNA by ABI Prism 7500 Detection System (Applied Biosystems, USA). GAPDH and U6 served as the internal control. The relative expression of RNA was calculated by the 2-*^ΔΔ^*ct method. The primer sequences are found in Table 1.

### 2.7. ELISA

Twenty-four hours after injection, the mice were anesthetized with sodium pentobarbital (30 mg/kg, intraperitoneal injection). After the mice were fixed, the eyeballs were picked with optical tweezers for blood collection. And culture supernatant of cells was collected. TNF-*α*, IL-1*β*, and IL-6 in samples were determined by the ELISA kit (Abcam) following the manufacturer's protocols. The absorbance at 450 nm was recorded using a microplate reader.

### 2.8. MTT Assay

Cells were incubated in a 96-well culture plate with 0.5 × 10^4^ cells/well for 48 h with 5% CO_2_ at 37 degrees. 20 *μ*l MTT (5 *μ*g/l, Boster, Wuhan) was then added in the wells and cultured for 4 h. After that, 150 *μ*l DMSO (Sigma-Aldrich) was added, and the absorbance at 490 nm was measured by a microplate reader for cell proliferation.

### 2.9. Flow Cytometry

Briefly, BEAS-2B cells were cultured in 6-well plates followed by the treatment with LPS and transfection. After 24 h, cells were collected, centrifuged, and resuspended in buffer solution. Annexin V/PI double staining (BD Biosciences, USA) was used to detect the apoptosis of cells, and then, cells were cultured in the dark for 15 min. BD FACS software (BD Biosciences, USA) was applied for quantifying the apoptosis ratio.

### 2.10. Western Blot

In short [26], the total protein was obtained by RIPA Lysis Buffer (Beyotime), separated, and transferred to PVDF membranes. Then, primary antibodies (Abcam) against Bcl-2 (1 : 000), Bax (1: 1000), cleaved caspase-3 (1 : 500), caspase-3 (1 : 500), IL2RB (1 : 500), and GAPDH (1 : 1000) were incubated on the membranes (4°C overnight). And then, horseradish peroxidase-conjugated secondary antibodies (1 : 3000) were incubated. All antibodies were purchased from Abcam. The results were visualized by ECL solution (Thermo Fisher Scientific).

### 2.11. Chromatin Immunoprecipitation (ChIP) Assay

EZ-Magna ChIP G Chromatin Immunoprecipitation Kit (Millipore, USA) was applied in this assay. In short, formaldehyde was used to treat cells for 10 min. The crosslinked chromatin was prepared and subsequently sonicated to fragments 200 to 400-bp in length, and were immunoprecipitated with IgG antibody (Negative control, Abcam) or SP1 antibody (Abcam) for 2 h (4°C). The precipitated chromatin DNA was eluted, reversed cross-links and treated with proteinase K before qRT-PCR analysis.

### 2.12. Statistical Analysis

GraphPad Prism 8.0 was applied to analyze the data. Each experiment was repeated in triplicate, and data were expressed as mean ± SD. The statistical significances were assessed by Student's *t* tests or one-way analysis of variance with the Tukey-Kramer post hoc test. *p* < 0.05 represented a significant difference.

## 3. Results

### 3.1. Bioinformatics Predicted miR-497-5p and the Target Genes

**Figure 1:**
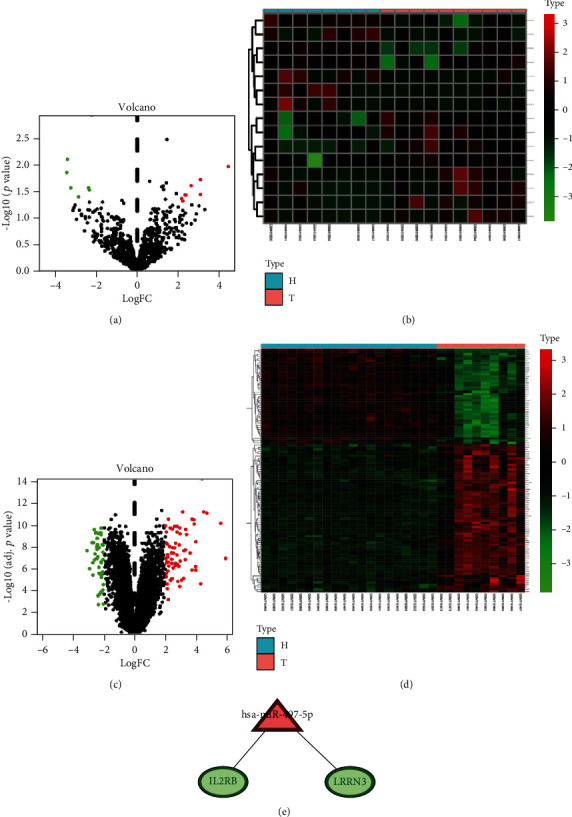
Bioinformatics predicted miR-497-5p and the target gene IL2RB. Volcano plots of DEMs (a) and heat map (b) analysis miRNA expressions in sepsis or corresponding normal samples from GEO database. Volcano plots of DEGs (c) and heat map (d) analysis mRNA expressions in sepsis or corresponding normal samples from GEO database. Volcano plot red dots: significantly highly expressed genes; green dots: significantly poorly expressed genes. Heat map red: significantly highly expressed genes; green: significantly poorly expressed genes. (e) The miRNA-target regulatory network. Triangles and circles represented miRNAs and target genes, respectively. Red represented genes that were upregulated, and green represented genes that were downregulated.

To determine the dysregulated miRNAs in sepsis, miRNA and mRNA profiles were obtained using the GEO database. Differentially expressed miRNAs (DEMs) and mRNAs (DEGs) were screened by the Limma package, and 15 DEMs and 110 DEGs are shown in Figures 1(a)–1(d). The target genes of DEMs were predicted using FunRich (version 3.13), and the intersection of the predicted target gene and DEGs was performed. Screened miRNA-target pairs were based on the relationship between miRNA and the target gene negative regulation (Figure 1(e)). The results show that 2 miRNA-target pairs were predicted. Among them, IL2RB had been proven to regulate inflammation in other diseases [22–24]. Hence, miR-497-5p/IL2RB was selected to validate its role in the alleviation of sepsis-induced ALI.

### 3.2. miR-497-5p Was Upregulated in LPS-Treated BEAS-2B Cells and Septic Mice

**Figure 2:**
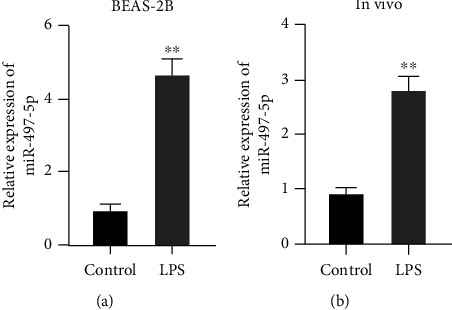
miR-497-5p was upregulated in LPS-treated BEAS-2B cells and septic mice. (a) qRT-PCR for miR-497-5p level in LPS-treated BEAS-2B cells. (b) qRT-PCR for miR-497-5p level in septic mice.

To explore the effect of miR-497-5p, LPS-treated mice or BEAS-2B cells were employed to construct sepsis-induced ALI *in vivo* and *in vitro* models, and miR-497-5p expression was tested. The results in Figure 2 showed that LPS treatment significantly increased miR-497-5p expressions of BEAS-2B cells and septic mice, illustrating that miR-497-5p was upregulated by sepsis and sepsis-induced inflammation.

### 3.3. Suppression of miR-497-5p Reduced LPS-Induced Apoptosis and Inflammatory Cytokine Production in BEAS-2B Cells

**Figure 3:**
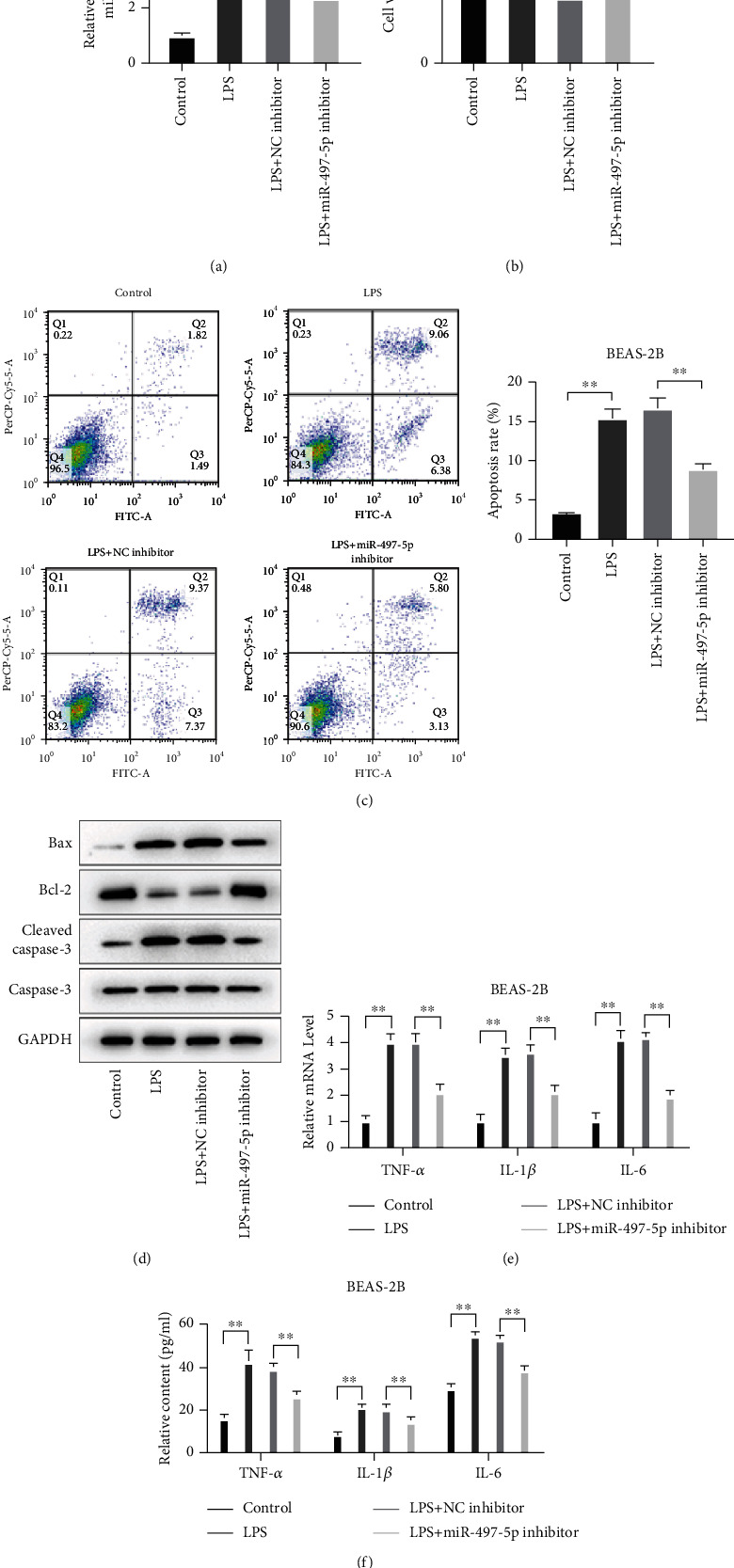
Suppression of miR-497-5p reduced LPS-induced apoptosis and inflammatory cytokine production in BEAS-2B cells. LPS was used to stimulate BEAS-2B cells, and then cells were transfected with miR-497-5p inhibitor. (a) qRT-PCR for miR-497-5p level detection. (b) MTT assay for cell viability detection. (c) Flow cytometry for the ratio of apoptosis. (d) Western blot for apoptosis-related protein expression. qRT-PCR and ELISA for mRNA level (e) and content (f) of TNF-*α*, IL-1*β*, and IL-6 detection.

For the purpose of studying the effect of miR-497-5p on ALI induced by sepsis, we synthesized the miR-497-5p inhibitor and its control for cell transfection after LPS treatment; miR-497-5p expression was remarkably reduced (Figure 3(a)). MTT and flow cytometry results showed decreased apoptosis and increased cell viability in LPS-treated BEAS-2B cells after miR-497-5p inhibitor transfection (Figures 3(b) and 3(c)). Meanwhile, western blot results also showed that the miR-497-5p inhibitor elevated Bcl-2 expression and lessened the expressions of Bax and cleaved caspase-3 in BEAS-2B treated with LPS (Figure 3(d)). In addition, the increase in the expression and content of TNF-*α*, IL-1*β*, and IL-6 caused by LPS treatment was verified by qRT-PCR and ELISA (Figures 3(e) and 3(f)) and was reduced after miR-497-5p inhibitor transfection. The above data suggested that miR-497-5p deficiency decreased apoptosis and attenuated the increase of inflammatory cytokines caused by LPS.

### 3.4. SP1 Regulated miR-497-5p Expression via Binding to Its Promoter

**Figure 4:**
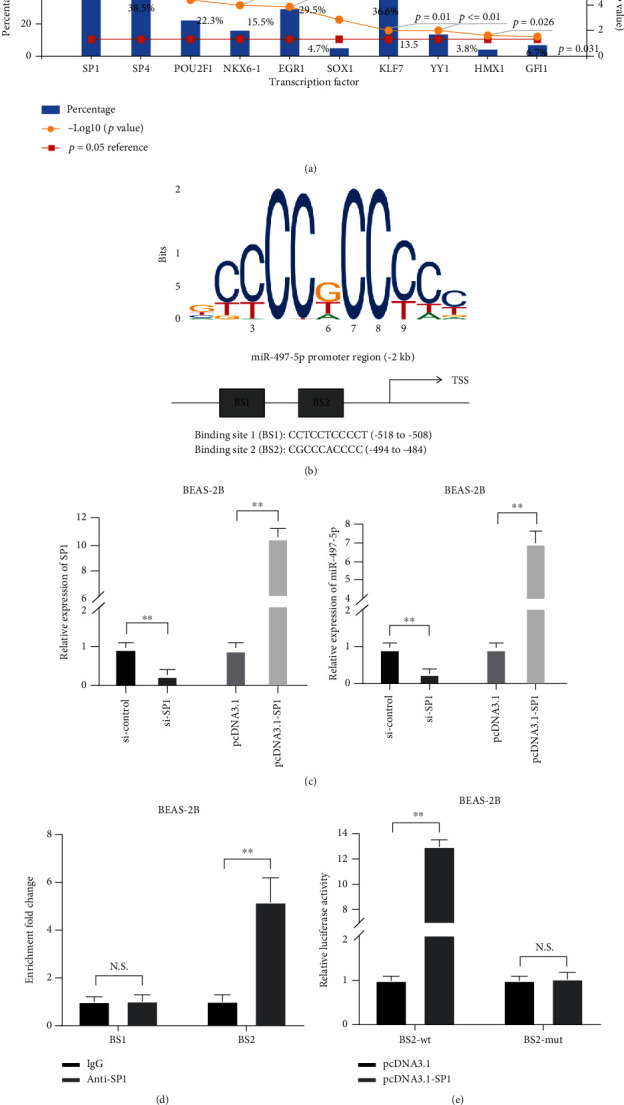
SP1 regulated miR-497-5p expression via binding to its promoter. (a) The expression of SP1 in the normal group and sepsis group in the database. (b) The dataset was used to predict the binding sites of SP1 in miR-497-5p promoter. (c) qRT-PCR for SP1 and miR-497-5p detection in BEAS-2B cells treated with si-SP1 or pcDNA3.1-SP1. (d) ChIP assay and (e) luciferase activity analyses for the SP1-binding site analysis.

To explore which transcript factor could play the key role in promoting miR-497-5p expression, we searched the database. The results showed that the SP1 level had the most significant difference between the control and sepsis groups among several transcription factors (Figure 4(a)). SP1-binding sites in the miR-497-5p promoter sequence are shown in Figure 4(b). Besides, qRT-PCR were applied to verify that si-SP1 was capable to reduce SP1 mRNA and miR-497-5p levels, while pcDNA3.1-SP1 elevated SP1 mRNA and miR-497-5p levels (Figure 4(c)). To determine the interaction, ChIP analysis was performed and the results revealed that, among the binding sites of the miR-497-5p promoter, the binding activity of SP1 at binding site 2 (BS2) was notably increased (Figure 4(d)). And luciferase activity analysis results validated that luciferase activities of cells with BS2 luciferase reporter plasmid transfection were remarkably increased by SP1 overexpression (Figure 4(e)). Overall, these above results validated that SP1 regulated miR-497-5p expression in BEAS-2B cells.

### 3.5. miR-497-5p Regulated Targeted Gene IL2RB Expression

**Figure 5:**
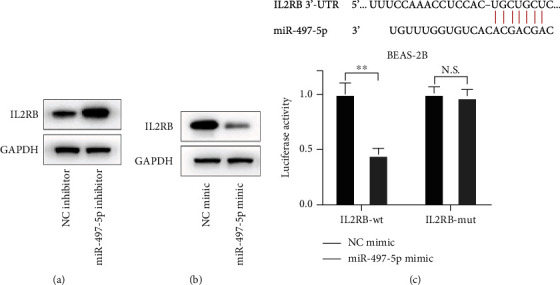
miR-497-5p regulated targeted gene IL2RB expression. (a) Cells were transfected with miR-497-5p inhibitor, western blot for IL2RB expression. (b) Cells were transfected with miR-497-5p mimic and western blot for IL2RB expression. (c) Cells were treated with IL2RB-wt or IL2RB-mut and miR-497-5p mimic; the luciferase activity was measured.

IL2RB reduced inflammation [27]. Western blot results displayed that miR-497-5p inhibitor or mimic transfection notably increased or inhibited IL2RB expression in BEAS-2B cells (Figures 5(a) and 5(b)). Additionally, the target of miR-497-5p was confirmed to be IL2RB, as evidenced by miR-497-5p mimic transfection inhibiting the luciferase activity of IL2RB-wt (Figure 5(c)). These results showed that miR-497-5p could target the expression of IL2RB.

### 3.6. miR-497-5p/IL2RB Was Essential for LPS-Induced Apoptosis and Inflammatory Cytokine Production in BEAS-2B Cells

**Figure 6:**
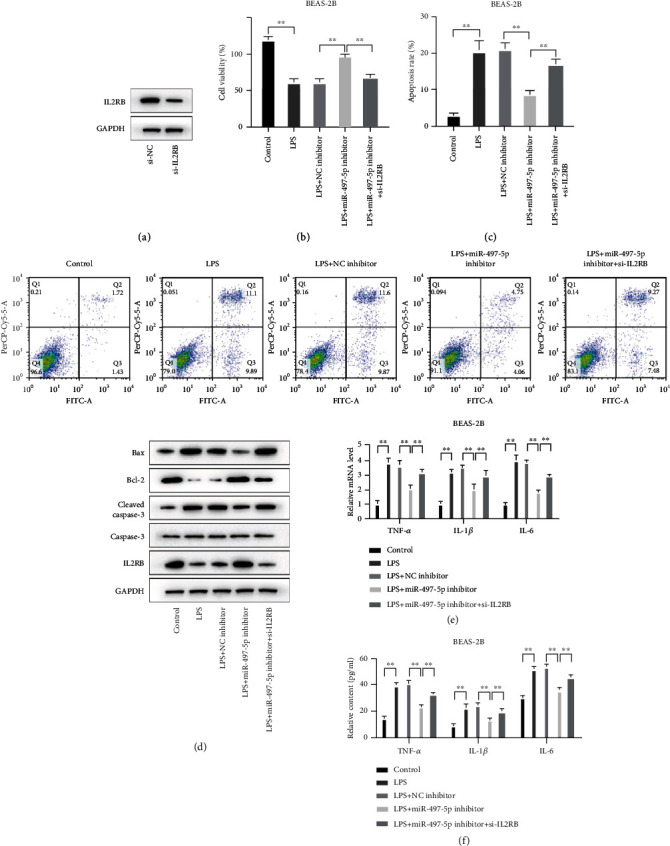
miR-497-5p/IL2RB was essential for LPS-induced apoptosis and inflammatory cytokine production in BEAS-2B cells. LPS was used to stimulate BEAS-2B cells, and BEAS-2B cells were treated with miR-497-5p inhibitor and si-IL2RB. (a) Western blot for IL2RB expression detection. (b) MTT assay for cell viability detection. (c) Flow cytometry for the ratio of apoptosis. (d) Western blot for apoptosis-related protein expressions. qRT-PCR and ELISA for mRNA level (e) and content (f) of TNF-*α*, IL-1*β*, and IL-6.

For further investigating whether miR-497-5p/IL2RB affects sepsis-induced ALI *in vitro*, we firstly transfected BEAS-2B cells with si-IL2RB to knock down IL2RB expression (Figure 6(a)). The results in Figures 6(b) and 6(c) represented that IL2RB deficiency reduced cell viability and elevated apoptosis in the miR-497-5p inhibitor and LPS-treated BEAS-2B cells. Western blot results also supported the above findings with the Bcl-2 expression dropped and the Bax and cleaved caspase-3 expression elevated (Figure 6(d)). Besides, si-IL2RB blocked the protective functions of miR-497-5p suppression against the LPS-caused inflammatory cytokine production (Figures 6(e) and 6(f)). Overall, miR-497-5p deficiency mitigated inflammatory cytokine production and apoptosis in LPS-treated BEAS-2B cells at least partly via IL2RB.

### 3.7. Downregulation of miR-497-5p Improved ALI Induced by Sepsis

**Figure 7:**
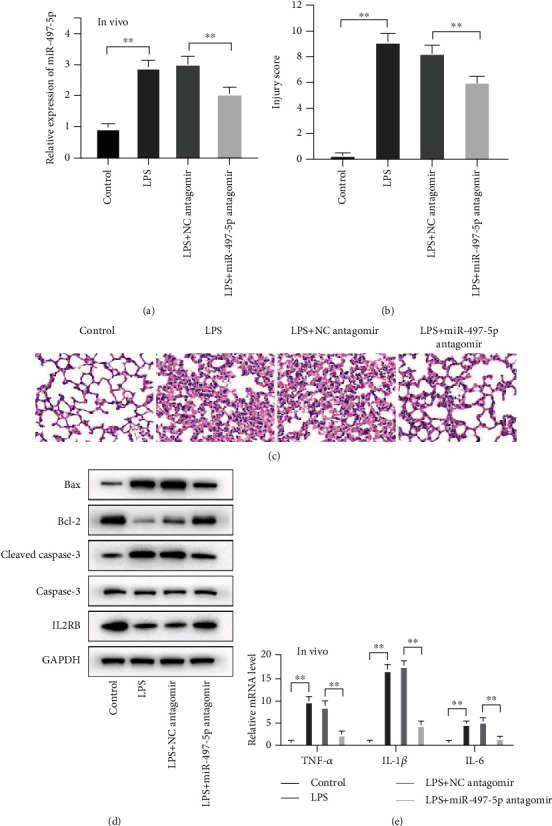
Downregulation of miR-497-5p improved ALI induced by sepsis. The septic mice with ALI were established by stimulating mice with LPS. The lung tissues of sacrificed mice were obtained. (a) qRT-PCR for miR-497-5p pulmonary expression. (b) H&E was used to stain sectioned lung tissue samples and lung injury scoring for the degree of lung injury. (c) H&E histological images (400x magnification). (d) Western blot for apoptosis-related protein expression. (e) qRT-PCR for mRNA levels of TNF-*α*, IL-1*β*, and IL-6.

Then, we studied the regulatory effect of miR-497-5p on the sepsis model by treating mice with LPS. Before LPS treatment, the intravenous injection of miR-497-5p antagomir was used to suppress miR-497-5p expression in septic mouse lung tissues (Figure 7(a)). And histological injury was mitigated by miR-497-5p antagomir (Figure 7(b)). H&E staining of lung sections showed that the morphological structure of pulmonary alveoli in sham-operated group was normal. However, lung sections of septic mice showed collapsed alveolar sacs, thickened alveolar walls and septa, visible vascular congestion, and hemorrhage. And the above-mentioned alveolus damage caused by sepsis could be alleviated by miR-497-5p antagomir injection (Figure 7(c)). Furthermore, we detected the apoptosis and inflammatory cytokine levels in the lung tissue of septic mice. We found that the miR-497-5p antagomir injection induced a significant increment of Bcl-2 expression and a reduction of Bax and cleaved caspase-3 expression in septic mice (Figure 7(d)). Meantime, the levels of inflammatory cytokine in septic mice were consistent with the above trends. miR-497-5p antagomir significantly suppressed the increase of inflammatory cytokine levels caused by LPS (Figure 7(e)). The survival rates of the sepsis-induced acute lung injury mouse model are shown in the Supplementary Material (available here). These findings, in conjunction with the above results, uncovered that low expression of miR-497-5p relieved apoptosis and inflammation, as well as protected mice from sepsis-induced ALI.

## 4. Discussion

Sepsis is a rapidly developing complication that is commonly associated with severe trauma, burns, and major surgery [28]. However, the excessive reaction of the body to external damage is the underlying cause of organ injury including acute lung injury (ALI) caused by sepsis [29]. Therefore, it is important to uncover the pathogenesis of reducing or inhibiting ALI. Here, we reported that downregulation of miR-497-5p could alleviate ALI caused by sepsis for the first time. Further studies had demonstrated that downregulation of miR-497-5p exerted the above-mentioned protective effects by negatively regulating the downstream target IL2RB. These conclusions were supported by the following evidence: the miR-497-5p-target relationship pairs were predicted in the sepsis database via bioinformatics. miR-497-5p was upregulated both in septic mice and LPS-treated BEAS-2B cells and had a negative correlation with the IL2RB level. The luciferase reporter gene experiment and ChIP analysis verified the binding relationship between miR-497-5p and IL2RB. The downregulation of miR-497-5p reduced sepsis-induced inflammatory cytokine production and apoptosis, while the downregulation of IL2RB attenuated this protective effect.

The most common pathogen of ALI was gram-negative bacteria. LPS on the bacterial surface was a key component that induced sepsis [30]. The LPS-induced ALI animal model reproduced the acute injury of lung tissue epithelium and acute alveolus inflammation through inhalation or exposure to LPS for a short time [31, 32]. Therefore, we established the septic mouse model and an inflammation cell model by treating with LPS. BEAS-2B cells were applied to construct a cell model for studying biological processes including apoptosis and inflammatory factor production [33–35]. Compared with the sham-operation group, LPS mice showed abnormal alveolar morphology. ALI induced by sepsis was closely related to inflammation and lung cell apoptosis [36]. The proinflammatory factors activated the inflammatory pathways and amplified the inflammatory cascade, leading to worsening lung injury. And the inflammatory cytokine overproduction damaged tissues and caused multiple organ failure [37, 38]. Cleaved caspase-3 expression was an important sign of apoptosis [39]. Studies have reported that cytokines (TNF-*α*, IL-10, and TGF-*β*) could regulate apoptosis by regulating the activity of caspase-8 in the death-induced signaling complex or changing death or survival factor levels, which control the Fas apoptotic pathway in sepsis [40]. And excessive apoptosis could cause massive loss of immune cells, which could lead to protracted inflammatory responses [41]. In this study, it was found that cleaved caspase-3 expression level enhanced TNF-*α*, IL-1*β*, and IL-6 levels, and cell apoptosis was elevated in LPS-treated mice and cells.

Recent researches have sported that miRNAs were related to regulate the initiation and development of the immune response to ALI in sepsis. miRNAs are expected to become biomarkers for the diagnosis and treatment of sepsis [42]. Based on targeting specific molecules or downstream genes, miRNAs regulated inflammation and apoptosis in the process of ALI [43]. miR-146 participated in the ALI inflammatory reaction process, with significantly increased expression levels in an ALI model in vitro [44]. miR-27a reduced inflammation and apoptosis by inactivating TLR4/MyD88/NF-*κ*B, thereby mitigating LPS-induced ALI in mice [45]. After targeting PGRN, miR-34b-5p deficiency alleviated the deterioration of lung apoptosis and inflammation in ALI mice induced by sepsis [46]. miR-539-5p reduced ALI caused by sepsis via targeting ROCK1 and showed the inhibitory effect of apoptosis and inflammation in MPVECs [47]. In our study, miR-497-5p levels were found to be elevated in LPS mice. The injection of miR-497-5p antagomir reduced apoptosis and inhibited the level of inflammatory factors, thereby effectively reducing ALI induced by sepsis. The low expression of miR-497-5p also showed the inhibitory effect of apoptosis and inflammatory cytokine production in BEAS-2B cells treated with LPS.

In our study, IL2RB was predicted and confirmed as the target gene of miR-497-5p. Here, the effect of knocking down miR-497-5p in reducing apoptosis and inflammatory factor levels in ALI was reversed by inhibiting IL2RB expression in BEAS-2B cells. By targeting upregulation of the IL2RB expression, low expression of miR-497-5p improved ALI caused by sepsis.

In conclusion, miR-497-5p deficiency alleviated the deterioration of sepsis-induced apoptosis and inflammation by upregulating IL2RB, which provided the basis for enriching the potential therapy of sepsis and ALI induced by sepsis.

## Figures and Tables

**Table 1:** The primer sequence used in qRT-PCR.

IL-1*β* (forward)	5′-CTCCGACCACCACTACAGCAAG-3′
IL-1*β* (reverse)	5′-TGGGCAGGGAACCAGCATC-3′
TNF-*α* (forward)	5′-CCCGAGTGACAAGCCTGTAGCC-3′
TNF-*α* (reverse)	5′-CCCTTGAAGAGGACCTGGGAGTAGAT-3′
IL-6 (forward)	5′-CAATGAGGAGACTTGCCTGGTG-3′
IL-6 (reverse)	5′-GCTGGCATTTGTGGTTGGG-3′
miR-497-5p (forward)	5′-CAGCAGCACTGTGGTTTGT-3′
miR-497-5p (reverse)	5′-CGACAGCAGCACACTGTGGTT-3′
SP1 (forward)	5′-TGGCAGCAGTACCAATGGC-3′
SP1 (reverse)	5′-CCAGGTAGTCCTGTCAGAACTT-3′
U6 (forward)	5′-CTCGCTTCGGCAGCACA-3′
U6 (reverse)	5′-AACGCTTCACGAATTTGCTTC-3′
GAPDH (forward)	5′-TCAAGGCTGAGAACGGGAAG-3′
GAPDH (reverse)	5′-TGGACTCCACGACGTACTCA-3-3′

## Data Availability

The datasets used and/or analyzed during the current study are available from the corresponding author on reasonable request.

## Supplementary Materials

Supplementary Materials Figure S1: sepsis mouse model was constructed by LPS, and the intravenous injection of miR-497-5p antagomir was used to suppress miR-497-5p expression in septic mice. The survival rates of mice in different treatments.
