# Supplementary material for: Downregulation of miR-497-5p Improves Sepsis-Induced Acute Lung Injury by Targeting IL2RB
Source: Biomed Res Int. 2021 Apr 12;2021:6624702. doi: 10.1155/2021/6624702 (PMC8057895; doi:10.1155/2021/6624702)
Supplement: Supplementary Materials — Figure S1: sepsis mouse model was constructed by LPS, and the intravenous injection of miR-497-5p antagomir was used to suppress miR-497-5p expression in septic mice. The survival rates of mice in different treatments. [file 6624702.f1.docx]

**Supplementary Material**

**
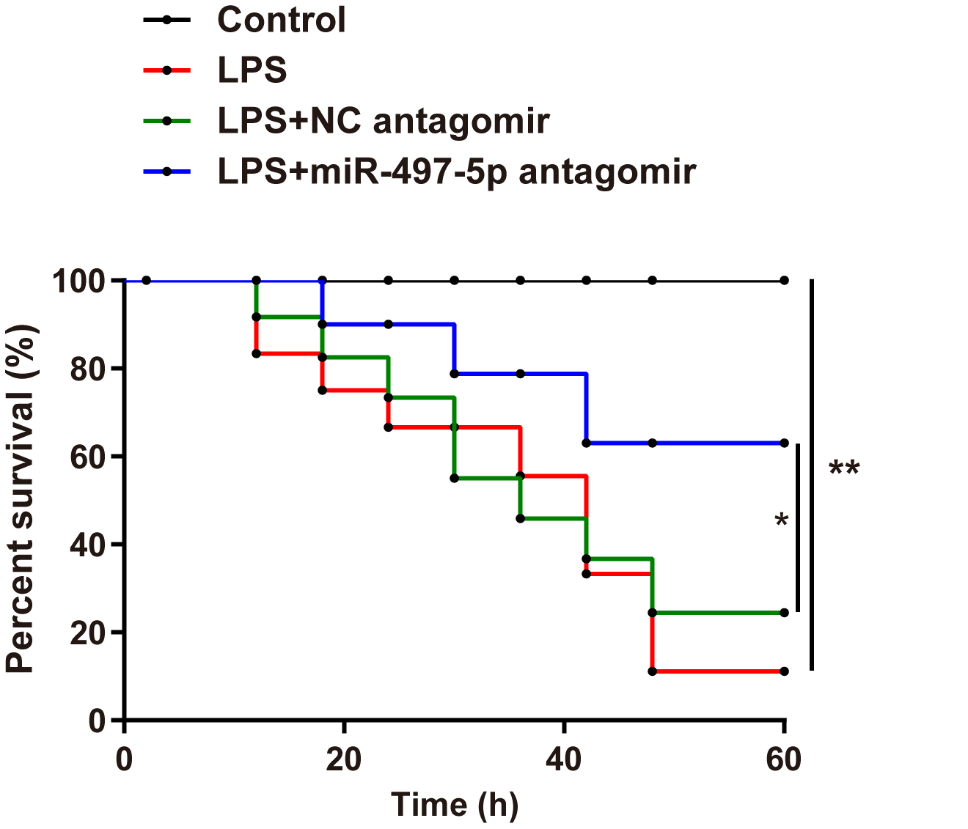
**

Figure S1. Sepsis mice model was constructed by LPS, and the intravenous injection of miR-497-5p antagomir was used to suppress miR-497-5p expression in septic mice. The survival rates of mice in different treatments.
